# Vestibular disorders in patients after COVID-19 infection

**DOI:** 10.3389/fneur.2022.956515

**Published:** 2022-09-20

**Authors:** Katarzyna Pazdro-Zastawny, Karolina Dorobisz, Paula Misiak, Anna Kruk-Krzemień, Tomasz Zatoński

**Affiliations:** Department of Otolaryngology, Head and Neck Surgery, Wroclaw Medical University, Wroclaw, Poland

**Keywords:** vestibular disorders, vertigo, dizziness, SARS-CoV-2, COVID-19

## Abstract

**Introduction:**

The COVID-19 clinical symptoms are primarily related to the respiratory system but may also be involved in many others, including the nervous system. Recently, vertigo or dizziness has been described as one of the clinical manifestations and possible complications of COVID-19.

**Materials and methods:**

This clinical study was designed to describe the otorhinolaryngological evaluation and videonystagmographic (VNG) findings in patients with an antecedent of COVID-19 infection in the last 6 months. In this study, we sought to investigate the presence of persistent vestibular damage in healed COVID-19 patients and to determine the origin of vertigo by conducting a comprehensive vestibular examination. To evaluate the association precisely, an otoneurological assessement was conducted on all participants. The study group included 58 patients aged 23–75 years with vertigo, who were diagnosed with COVID-19 infection 6 months before the examination. Each participant was submitted to an evaluation consisting of anamnesis, otorhinolaryngological evaluation, and VNG.

**Results:**

Spontaneous nystagmus with closed eyes was reported in 8 patients (13.8%). Positional nystagmus was observed in 15 patients (24.1%). Asymmetrical optokinetic nystagmus was observed in 18 patients (31%). A distorted record in the tracking pendulum test was present in 23 patients (39.7%). Square waves were observed in 34 COVID-19 patients (58.6%). Unilateral weakness (UW) was observed in 23 subjects (39.7%); among those with UW, 22 patients (95.7%) also demonstrated directional preponderance contralateral to the UW. Another 16 patients (27.6%) presented only directional advantage. The post-caloric recruitment was present in 38% patients.

**Conclusion:**

Patients who had been diagnosed with COVID-19 seem to be more likely to suffer from vertigo/dizziness and to compensate more slowly. COVID-19 infection may cause inner ear damage and lead to vestibular dysfunction. The role of the central nervous system in the onset of equilibrium disorders should be considered. The presence of vertigo of central origin may indicate the neurotropic effect of SARS-CoV-2 following COVID-19. Imbalance may be the only symptom of COVID-19 and may also be a late complication of the disease due to post-infectious inflammation of the nervous tissue. Comprehensive studies are needed to investigate whether COVID-19 can cause long-term vestibular deficits.

## Introduction

The COVID-19 pandemic caused by the coronavirus SARS-CoV-2 is currently a major public health problem worldwide. The clinical symptoms are primarily related to the respiratory system, but may also be involved in many others, including the cardiovascular, immune, digestive, urinary, and nervous systems. COVID-19 can cause acute, long-term neurological complications in both the initial stages of the disease and the protracted recovery period. The neurological manifestations can be nonspecific symptoms—such as headache, altered mental status, insomnia, anxiety, depression, or disturbances of consciousness, confusion, epileptic seizures, myalgia—or more specific symptoms and syndromes requiring immediate medical attention (1). There are studies in the literature that evaluated the impact of COVID-19 on the incidence of cochlear-vestibular disorders (2, 3). Recently, neurological involvement presenting as vertigo or dizziness has been described as a clinical manifestation and possible complication of COVID-19. Mao et al. revealed that apart from the typical general and respiratory symptoms, 36.4% of patients with COVID-19 had neurological symptoms, including headaches, disturbances of consciousness, and paresthesia. Patients with a severe course of the disease are at greater risk of developing neurological disorders, including acute cerebrovascular diseases, impaired consciousness, and skeletal muscle injury than patients with mild to moderate symptoms (4).

### Study design

The aim of this study was to describe the otorhinolaryngological evaluation and videonystagmographic (VNG) findings in patients with an antecedent of COVID-19 infection in the last 6 months. In this study, we sought to investigate the presence of persistent vestibular damage in healed COVID-19 patients and to determine the origin of vertigo by conducting a comprehensive vestibular examination.

The study group included 58 patients aged 23 to 75 years (mean age: 48 years) with vertigo who were diagnosed with COVID-19 infection 6 months before the examination. Women constituted the majority of the patients (56.9%). Baseline demographic and clinical characteristics of the study group are presented in Table 1. The prevalence of symptoms reported by recovered COVID-19 patients with vertigo is shown in Table 2.

**Table 1:** Baseline demographic and clinical characteristics of the study group.

**Study group (*****N*** = **58)**		
Females, *n* (%)	33	(56.9)
**Vertigo**		
Rotational*, n* (%)	13	(22.4)
Dizziness*, n* (%)	21	(36.2)
Both*, n* (%)	4	(6.9)
Vertigo circumstances^*^		
Associated with fear, *n* (%)	4	(6.9)
Associated with a change in body position, *n* (%)	19	(32.8)
During head rotation, *n* (%)	11	(19.0)
Associated with fatigue, *n* (%)	6	(10.3)
After physical activity, *n* (%)	6	(10.3)
No obvious reason, *n* (%)	12	(20.7)
Other, *n* (%)	2	(3.4)
**Duration of vertigo**		
Seconds, *n* (%)	16	(27.6)
Minutes, *n* (%)	9	(15.5)
Few hours, *n* (%)	5	(8.6)
Days, *n* (%)	6	(10.3)
**Vertigo frequency**		
Everyday, *n* (%)	21	(36.2)
Once a week, *n* (%)	7	(12.1)
Few times a week, *n* (%)	4	(6.9)
Few times a year, *n* (%)	6	(10.3)
One episode only, *n* (%)	2	(3.4)
Other symptoms^*^		
Nausea, *n* (%)	7	(12.1)
Vomiting, *n* (%)	9	(15.5)
Palpitations, *n* (%)	9	(15.5)
Drench sweats, *n* (%)	7	(12.1)
Fainting, *n* (%)	2	(3.4)

n, number of participants; (%), percentage;

* multiple-choice question (proportions do not add up to 100).

**Table 2:** Prevalence of symptoms reported by recovered COVID-19 patients with vertigo.

**Additional symptoms**		
Olfactory disorders, *n* (%)	10	(17.2)
Ocular disorders, *n* (%)	20	(34.5)
Taste disturbances, *n* (%)	8	(13.8)
A tingling feeling in the face, *n* (%)	4	(6.9)
Abnormal facial expression, *n* (%)	5	(8.6)
Difficulty with swallowing and/or hoarseness, *n* (%)	9	(15.5)
Increased tension of the face, neck, neck pain, *n* (%)	24	(41.4)
Head trauma, *n* (%)	11	(19.0)

The inclusion criteria were patients who presented with vertigo, were at least 18 years of age, had recovered from COVID-19 infection with a mild to moderate course, were confirmed positive by polymerase chain reaction (PCR) test, and were referred to the vestibular service of our institution in an ambulatory clinic. The exclusion criteria were patients with previous otological or neurological problems; hospitalization for COVID-19; a previous history of dizziness/vertigo; and psychiatric, cardiovascular, or circulatory comorbidities. One patient with a history of intensive care unit hospitalization was excluded from the study.

## Methods

Each participant underwent an evaluation consisting of anamnesis, otorhinolaryngological evaluation, and VNG. The anamnesis involved a detailed otoneurological interview on the patients' clinical history with particular attention paid to the current disease and the presence of vestibular and balance disorders. The patients were asked about their main ailments, chronic and past diseases—particularly pathologies of the middle and inner ear—COVID-19 diagnosis, and any conditions that may affect the vestibular organ, dizziness, and balance disorders. Attention was paid to symptoms that could indicate vestibular organ disorders, such as unstable body posture; timeline; and exacerbating factors of dizziness.

All subjects were examined with VNG, which included calibration and registration of the following:

- possible spontaneous nystagmus with open/closed eyes
- positional nystagmus with eyes closed in four positions: supine, on the right side, on the left side, and on the back with the head tilted back (Rose position)
- after performing Hallpike maneuvers to the right and left, the assessment of positional nystagmus (PN) was based on the classification proposed by Nylen: Nylen I (PN I)—nystagmus changing direction with a change of head position; Nylen II (PN II)—nystagmus showing a constant direction of the fast phase regardless of the head position; Nylen III (PN III)—irregular nystagmus, manifested by significant variability in both the direction and magnitude of the amplitude and frequencies, sometimes alternating in the same head position
- optokinetic nystagmus to the right and left; the recording was rated as symmetrical (correct) or asymmetrical according to the course and average velocity of the free phase of nystagmus
- alternate binaural bithermal caloric test with cool (30°C) and warm air (44°C) irrigations of 30 s each
- tracking pendulum test (a moving point of light displayed on the screen with a frequency of 0.4 Hz and an amplitude of 30°, alternately clockwise and counterclockwise, was observed by the subject; the speed of the light point was 24°/s. The recording of continuous eye movements was 30 s; the obtained record was assessed qualitatively as normal in the case of a smooth tracking curve or as incorrect and distorted.)

Nystagmus assessment was performed by the computer system evaluating the frequency of nystagmus deflections in selected 30 s of the peak of reaction. Air caloric stimulation is the routine test battery of our otoneurological department, even in cases of normal external ear canal and normal tympanic membrane. Jongkees's formula was used to assess nystagmus symmetry. The following norms for caloric test parameters were selected (5):

- unilateral weakness: <25%
- directional preponderance (DP): <30%.

In several cases, central vertigo is accompanied by oculomotor signs, including changes in saccades and in pursuit movements. Peripheral vestibular disease was diagnosed in case of the absence of signs of central nervous system impairment, and the caloric test values of UW and DP were higher than the normal parameters.

The post-caloric recruitment index (PCRI) was defined based on the Jongkees formula, comparing the results of the bi-thermal AVSP test: PCRI = (cold AVSP–warm AVSP/cold AVSP+warm AVSP) × 100. The established normal value was PCRI ≤ 17% (6).

The vestibular testing was conducted by a trained examiner who had no knowledge of the subjects' health.

Permission for the subjects to participate in this study was approved and granted by the Local Ethics Committee of the Medical University in Wrocław, Poland. All participants took part in the study voluntarily and were able to discontinue their participation at any time without repercussion. The participants were informed of the nature of the study; in each case, they formally expressed their consent to participate. The study was performed in accordance with the ethical standards laid down in the 1964 Declaration of Helsinki and its later amendments.

### Statistical analysis

Statistical analysis was performed using STATISTICA v. 13.3 (TIBCO Software Inc., Palo Alto, CA, USA). The distributions of quantitative parameters were checked against a normal distribution using the Shapiro–Wilk test. Depending on the distribution, the quantitative variables are presented in tables and graphs as means (M) and lower (Q1) and upper (Q3) quartiles.

## Results

The VNG revealed many abnormalities in COVID-19 patients. Spontaneous nystagmus with closed eyes was reported in 8 patients (13.8%). Positional nystagmus was observed in 15 patients (24.1%): PN I in 8.6%, PN II in 10.3%, and PN III in 5.2%. Asymmetrical optokinetic nystagmus was observed in 18 patients (31%). A distorted record in the tracking pendulum test was present in 23 patients (39.7%). Square waves were reported in the VNG recording in 34 COVID-19 patients (58.6%). UW was observed in 23 subjects (39.7%); among those with UW, 22 patients (95.7%) also demonstrated DP contralateral to the UW. Another 16 patients (27.6%) presented only DP. Bilateral vestibular areflexia syndrome was not observed. The post-caloric recruitment was present in 38% of patients. Among those with UW, the percentage of PCRI >17% was in 47% (11 patients). The UW patients with DP contralateral to UW presented also PCRI >17%. Table 3 presents the results of vestibular tests in recovered COVID-19 patients with vertigo.

**Table 3:** Results of vestibular tests in recovered COVID-19 patients with vertigo.

**Nystagmus**	**Study group**	
	**N** = **58**	
**Spontaneous nystagmus**		
Eyes close, *n* (%)	8	(13.8)
**Positional nystagmus**		
Nylen I, *n* (%)	5	(8.6)
Nylen II, *n* (%)	6	(10.3)
Nylen III, *n* (%)	3	(5.2)
Distorted tracking pendulum test, *n* (%)	23	(39.7)
Presence of dysmetry, *n* (%)	23	(39.7)
Presence of dysrhythmia, *n* (%)	21	(36.2)
Presence of square waves, *n* (%)	34	(58.6)
Asymmetrical optokinetic nystagmus, *n* (%)	18	(31.0)
Bithermal caloric tests with abnormal findings, *n* (%)	21	(36.2)
Unilateral weakness, *n* (%)	23	(39.7)
Unilateral weakness and contralateral directional preponderance, *n* (%)	22	(95.7)
Directional perponderance without unilateral weakness, *n* (%)	1	(4.3)
Directional preponderance, *n* (%)	16	(27.6)

n, number of participants; (%), percentage.

## Discussion

The vestibular organ is a complex, anatomically disperse (consisting of peripheral and central components) but functionally coherent system that maintains a person's balance through the simultaneous interaction of various subsystems. Vertigo is most often caused by a dysfunction in the vestibular system, from a peripheral or central lesion.

Observations suggest the possibility that SARS-CoV-2 can invade the neural pathways involved in balance. Due to the neurotropic and neuroinvasive properties of coronaviruses, neurological manifestations concerning the central nervous system (CNS) and the peripheral nervous system have been reported in hospitalized patients with COVID-19 (2). SARS-CoV-2 neurotropism may influence a broad range of neuropathic impact, including effects on the neural circuit that govern balance (7). In a 10-item, closed-ended questionnaire in Italian COVID-19 patients, subjective otoneurological symptoms such as tinnitus and balance disorders were observed. Out of the 18.4% of subjects who had balance disorders after a diagnosis of COVID-19, 94.1% reported dizziness and 5.9% reported acute vertigo attacks (8). In another study on patients with positive PCR test results for COVID-19, 31% of them reported nausea/vomiting (31%). Otological/vestibular symptoms were observed in the following proportions: dizziness (31.8%), tinnitus (11%), and true vertigo (6%) (9).

Any pathology affecting the vestibular nuclei or their projections—especially those to and from the cerebellum—can result in symptoms of vertigo and related nystagmus. Central vertigo can occur as a result of any lesion or dysfunction of the brainstem vestibular apparatus. Peripheral vertigo may be a result of problems in the peripheral vestibular system, from the inner ear to the vestibular division of the eighth cranial nerve. In central vertigo, abnormalities of the vestibular structures in the CNS result in hallucinations of motion of individual surroundings or a sensation of spinning despite remaining still. The patients typically complain of dizziness with hallucination or a sense of spinning. Oculomotor alterations are related to central vertigo, while UW in the caloric test is related to peripheral vertigo. Central vertigo is in most cases accompanied by oculomotor signs, including saccadic pursuit.

Vertigo of central origin was observed in 53.4% of the patients. An abnormal VNG recording in the tracking pendulum test was observed. Square waves, dysrhythmias, and asymmetrical optokinetic nystagmus were also found in the VNG recordings in COVID-19 patients. The distortion of optokinetic nystagmus occurs as a result of damage to the CNS. In this study group, the frequency of PN III was 5.2%. This may indicate that etiopathology of vertigo/dizziness reported by patients is of central origin, which may be associated with the neurotropic and neuroinvasive properties of SARS-CoV-2 and may indicate multilevel damage to the balance system.

Many viral infections are known to cause immediate or delayed neuropathological changes and neurological manifestations, including anosmia, facial nerve paralysis, or sudden sensorineural hearing loss. In a study by Mao et al. (4), 16.8% of the patients reported dizziness. The authors stated that reported CNS symptoms were the main form of neurological injury in patients with COVID-19, suggesting that the pathological mechanism may be from CNS invasion.

The pathophysiology of CNS involvement has yet to be fully described. The neurotropic potential of SARS-CoV-2 may be direct, connected with the intranasal route of viral transmission through the olfactory nerves into the brain, and indirect, by damaging endothelial cells and pericytes, or *via* activation of neuroimmune responses. Inflammatory response of nervous tissues (10, 11) can lead to injury of blood vessels in the brain and brain cells (12–14) through the mechanism of the microglia (15–18). According to Margo et al. (13), one of COVID-19's pathomechanisms is the connection of pseudovirions to angiotensin-converting enzyme 2 (ACE2)^+^ endothelial cells, located in the brain, for example. ACE2 was identified as a SARS-CoV-2 functional receptor. SARS-CoV-2 binds to the ACE2 receptors once it enters the CNS. Likewise, the spike protein of SARS-CoV-2 may interact with ACE2 expressed in the capillary endothelium; thus, the virus may cross the blood–brain barrier and enter the CNS (19). Activation of the complement pathway/coagulation cascade leads to a systemic procoagulant state and the expression of cytokines that create a hypercoaguable state known as cytokine storm. Patients with severe COVID-19 are more prone to higher fibrin degradation product levels (D-dimer) and have a higher likelihood of cerebrovascular disease.

Spontaneous nystagmus with eyes closed was recorded in 13.8% of patients, which indicates that vestibular disturbances were not compensated. The other participants of the study did not report nystagmus. This may indicate that the patients experienced a subclinical course of the disease or had adapted to the vestibular disturbances. SARS-CoV-2 may influence the vestibular system through a mechanism of directly impairing inner ear structures or through a virus-triggered dysregulation of the immune system, with inflammation spreading to the cochlea (19).

According to Jongkess (20), DP is a tendency for more intense nystagmus in one direction than the other. However, its clinical significance is controversial. It is commonly recognized that DP is associated with the vestibular compensation process. DP has been observed in disorders of central and peripheral vestibular systems. In young patients with unilateral peripheral deficit syndrome, preserved vision, and proprioception, DP tends to disappear as soon as compensation is complete, although the process of vestibular compensation varies. Vestibular compensation is the rehabilitation of vestibular function after a unilateral vestibular loss. It is a rapid vestibulo-centric static process and a longer term, dynamic, distributed learning process. Vestibular recruitment is a mark of hyperexcitability of central vestibular neurons and may be specific to peripheral vestibular damage. The dynamics of the compensation process depends mainly on the efficiency of the CNS and the speed of the creation of new links between the vestibular organs and on the efficiency of vision and the sense of deep feeling. Post-caloric recruitment is a combination of peripheral and central effects. PCRI is the ratio of the angular velocity of the slow phase obtained by cold and warm caloric stimulation of the same ear. Recruitment indicates that the stimulus intensity and the response of the ocular motor neurons are regulated by central modulation. The recovery of peripheral vestibular dysfunction may be incomplete or complete. Imate et al. evaluated long-term vestibular function in vestibular neuronitis patients and found that there are three possibilities: (1) a change in complete vestibular compensation; (2) incomplete vestibular compensation; or (3) a change in peripheral vestibular dysfunction (21). The results of our study support the lack of adequate compensation at UW. These findings agree with the hypothesis that COVID-19 may cause a simultaneous peripheral and central vestibular damage. The recovered COVID-19 patients seemed to compensate slowly or in an improper way.

In our study, adequate vestibular compensation did not apply to most patients who presented vestibular UW. Even not precisely controlling the post-COVID-19 time to submit patients to the otoneurological battery, a contralateral directional preponderance to the UW and a PCRI > 17% among the majority of UW patients seems to indicate that the post-COVID-19 central balance compensation does not occur as usual. With more detailed studies, we will be able to assess whether vestibular deficits are temporary or permanent in post-COVID-19 patients.

PN occurs in certain head positions or its parameters change depending on the position of the head. Three types of PN have been distinguished: PN I is usually of central origin, PN II is caused by a peripheral canal disorder, and PN III is a result of a central lesion and is a combination of brief nystagmus and vertigo. PN II was present in 10.3% of the study group. PN I and PN III were present in 8.6 and 5.2% of the patients, respectively. The commonly accepted opinion is that PN II is mainly labyrinthiform. The most common cause of vestibular disturbances in adults is benign paroxysmal positional vertigo (BPPV) (1). The pathophysiological mechanism of BPPV is based on “cupulolithiasis” and “canalolithiasis” theories and may be a result of inner ear ischemia. Lesions are often found in the cerebellum and brainstem in central paroxysmal positional vertigo. The condition is predictive of lesions in the posterior fossa, involving a communicating network between the vestibular apparatus (otolith organs and semicircular canals), brainstem vestibular nuclei, and midline cerebellar structures within the vermis (22). BPPV is also associated with a viral infection, which might promote BPPV attacks due to the development of vestibulopathy or induce secondary BPPV *via* viral infection-related neurolabyrinthitis (23).

In our study, unilateral vestibular weakness was observed in 39.7%, abnormal results of the caloric test were present in 36.2%, and changes in oculomotor movements in around 40% (Table 3). These findings disclose an unexpected degree of neurological alterations after a vestibular disease post-COVID-19, when compared to the general population that presented a vestibular disease (24, 25). A study in the adult US population found a prevalence of vestibular hypofunction of 28/100,000 (26). In a descriptive study by Felipe et al., patients with body balance disorders revealed peripheral disease in 40.1% and central disease in 2.5% individuals. Abnormal results in the caloric test were found in 42.6%, with UW present in 31.7% patients with vertigo (24). The other study that analyzed VNG in patients with dizziness, vertigo, and balance disorders revealed vestibular disorders in 35.86% patients, benign paroxysmal positional vertigo in 18.9% of patients, mixed vertigo in 16.12% of patients, cervical vertigo in 5.42% of patients, vertigo and dizziness of central origin in 3.78% of patients, vascular vertigo in 2.8% of patients, and vestibular neuritis in 2.3% of patients (25). Over 25% of symptomatic older adults can be expected to have vestibular hypofunction. This is connected with the degenerative process involving the vestibular system, likely to be due to environmental factors such as exposure to infections (27).

COVID-19 infection is known to induce a vascular compromise of the neural tissue. Balance disorders can be related to vascular damage because the inner ear structures are particularly vulnerable to ischemia due to their terminal vasculature. Malayala et al. (28) presented vestibular neuritis cases in 4 positive and 2 suspected COVID-19 patients. However, only 2 patients presented nystagmus, which brings into question the connection between vestibular system disturbances and viral infection (29). Aljasser et al. revealed that rotatory vertigo, which could be vestibular in origin, may be a clinical manifestation of COVID-19. Vestibular disturbances were found in 5% of the COVID-19 groups and in 1.1% of the controls. Some patients (17.7%) reported unsteadiness or light-headedness following COVID-19 diagnosis. Vestibular system dysfunction may be a result of neuritis caused by viral invasion of the inner ear or the vestibulocochlear nerve, potentially leading to vertigo or accidental damage to the inner ear by antibodies or T cells misidentifying inner ear antigens as a virus (30). Also, vascular disorders or immune-mediated sequelae of immune-mediated disorders (excessive production of proinflammatory cytokines) may negatively affect the audio-vestibular system (18).

In COVID-19 patients, vertigo was symptomatic and was associated with anxiety in 6.9% of patients. Anxiety and stress are common during the COVID-19 pandemic; they can provoke vertigo attacks (31–33).

Our study revealed that vestibular symptoms were not transient and had not resolved at the time of screening in recovered COVID-19 patients. Health professionals should be aware that COVID-19 infection may result in vestibular disturbances and that such patients should be investigated and managed carefully. A healthcare management policy for dizziness/vertigo should be developed during the outbreak of COVID-19. Otoneurological assessment should be considered because vertigo is a prevalent neurological symptom after COVID-19 infection. Vertigo can severely impact long-term outcomes and can have a negative effect on post-COVID-19 quality of life. There is a crucial need for prompt screening and otoneurological evaluation in order to recognize vestibular disorders and introduce effective treatment.

## Strengths and limitations

In this descriptive study, the otorhinolaryngological evaluation and VNG findings in patients with an antecedent of COVID-19 infection in the last 6 months were examined. The strength of the study is the complete clinical otoneurological evaluation of the study participants, as self-reported measures may be unreliable. In our study, the role of CNS in the onset of symptoms was determined. The high prevalence of central vertigo in post-COVID-19 patients may indicate that such disturbances should be considered as a CNS COVID-19 complication.

One of the limitations of the study is the lack of evaluation of time between the COVID-19 infection and otoneurological examination. However, the analyzed sample size and found data enabled us to conclude that vestibular compensation did not seem to have occurred as usual in post-COVID-19 patients. The other limitation includes the lack of consistent follow-up of our patients, which limits us from enquiring into the recovery time of the reported symptoms, or from determining whether vertigo is a long-lasting ailment. Further studies should focus on establishing a better association between COVID-19 patients with vestibular disturbances and COVID-19 patients without a history of vertigo. Furthermore, we have recognized the need for further studies to evaluate mild/moderate and severe COVID-19 infection in relation to the otoneurological manifestations. Also, both mild COVID-19 infection and vestibular pathology are highly prevalent, so it could lead to a false conclusion of casualty.

## Conclusion

The COVID-19 infection may damage the inner ear and may lead to vestibular dysfunction that progresses with slower than expected compensation. The role of the CNS in the onset of equilibrium disorders should be considered. The presence of vertigo of central origin may indicate the neurotropic effect of SARS-CoV-2 following COVID-19. It might suggest multilevel damage to the balance system. Imbalance may be the only symptom of COVID-19, and it may be a late complication of the disease due to post-infectious inflammation of the nervous tissue. Comprehensive studies are needed to investigate whether COVID-19 can cause long-term vestibular deficits. Further evaluation of the equilibrium system in patients after COVID-19 is needed. In addition, studies are necessary to possibly correlate audio-vestibular symptoms with SARS-CoV-2 infection and to investigate the prevalence and pathophysiological mechanisms underlying these symptoms in COVID-19 patients.

## Data availability statement

The raw data supporting the conclusions of this article will be made available by the authors, without undue reservation.

## Ethics statement

The studies involving human participants were reviewed and approved by Local Ethics Committee of the Medical University in Wroclaw, Poland. The patients/participants provided their written informed consent to participate in this study.

## Author contributions

KP-Z and KD made substantial contributions to analysis and interpretation of data and have been involved in drafting the manuscript and revising it critically for important intellectual content, given final approval of the version to be published, and agreed to be accountable for all aspects of the work in ensuring that questions related to the accuracy or integrity of any part of the work are appropriately investigated and resolved. PM and AK-K made substantial contributions to analysis and interpretation of data, given final approval of the version to be published, and agreed to be accountable for all aspects of the work in ensuring that questions related to the accuracy or integrity of any part of the work are appropriately investigated and resolved. TZ made substantial contributions to conception and design and been involved in revising the manuscript critically for important intellectual content, given final approval of the version to be published, and agreed to be accountable for all aspects of the work in ensuring that questions related to the accuracy or integrity of any part of the work are appropriately investigated and resolved. All authors read and approved the final manuscript.

## Conflict of interest

The authors declare that the research was conducted in the absence of any commercial or financial relationships that could be construed as a potential conflict of interest.

## Publisher's note

All claims expressed in this article are solely those of the authors and do not necessarily represent those of their affiliated organizations, or those of the publisher, the editors and the reviewers. Any product that may be evaluated in this article, or claim that may be made by its manufacturer, is not guaranteed or endorsed by the publisher.
